# Self-care as self-preservation: where is the support for coaches' self-care in Canadian sport?

**DOI:** 10.3389/fspor.2023.1278093

**Published:** 2023-10-12

**Authors:** Emily McCullogh, Parissa Safai

**Affiliations:** School of Kinesiology and Health Science, Faculty of Health, York University, Toronto, ON, Canada

**Keywords:** coaches, care, self-care, safe sport, athletes, Canadian sport

Caring for myself is not self-indulgence, it is self-preservation, and that is an act of political warfare (1).

Sport is once again facing a crisis within Canada as, in recent months, increased reporting of athlete harm has further energized scholars’, policymakers', and practitioners' attention and efforts towards prioritizing safe sport policies and practices [e.g., see (2–6)]. This attention is firmly (and rightly) set on athletes' welfare; however, there remains great need to critically examine and restructure the conditions under which abusive and uncaring coaching behaviours are practiced [e.g., see (7)], including the neglect of care for coaches in the name of athlete development and performance. The absence of meaningful and sustained efforts towards supporting coaches' care is where we situate our work and prompts us to consider renowned Black queer feminist, academic, and civil rights activist Audre Lorde's oft-cited words about care: “Caring for myself is not self-indulgence, it is self-preservation, and that is an act of political warfare” (1).

We draw on these words as an entry point into a discussion about care for coaches (including their own self-care), or lack thereof, in the Canadian sport system. Our goals in this paper are twofold: (1) highlight how little attention is being paid to coaches’ care even though they are expected to care for others [cf. (7),]; and (2) issue a call-to-action to sport and sport coaching governing bodies to hold coaches' care as a fundamental requirement of the safe sport movement and to ensure, with transparency and accountability, that sport coaches are cared for and adequately resourced to engage in care/self-care practices.

The ways in which care and caring are conceptualized and practiced in the existing sport system prioritizes care for some over others and, thus, renders wellness privilege as invisible, individualized, and depoliticized. Too little attention has been paid to coaches' care and this gap is a political issue that requires collective response and action. If we are to meaningfully address and reduce the prevalence of athlete harm, we must stop thinking of care as a wellness issue reserved only for athletes but as a site and practice of power that is neither possessed by nor equally accessible to all sport participants at all times.

## Self-care as a political act

Following her second cancer diagnosis, Lorde published *A Burst of Light* (1988) in which she wrote of her cancer journey and mortality, alongside continued reflections on, and calls for, radical action to address deeply rooted social inequities arising from neoliberal, capitalist, racist, and sexist structures, systems, and power relations. Although this specific quote has come to dominate public consciousness, what Lorde wrote preceding this one sentence is illuminating:

I had to examine, in my dreams as well as in my immune-function tests, the devastating effects of overextension. Overextending myself is not stretching myself. I had to accept how difficult it is to monitor the difference. Necessary for me as cutting down on sugar. Crucial. Physically. Psychically. Caring for myself is not self-indulgence, it is self-preservation, and that is an act of political warfare. (p. 332)

For Lorde, self-care was never just an individual act in and of itself but rather inextricably linked to the health and well-being of others (9). This aligns with many other scholars and advocates (feminist or otherwise) who argue that self-care is not individualized pampering but is personal, political, and central to collective resistance against the systemic conditions that create and maintain inequity and harm for individuals and groups [cf., (10); see also (11–14)].

It is important to note that Lorde's words have been seized upon to support a multi-billion dollar global “cult of wellness” (15) that “…reinforces the very structures Audre Lorde devoted her life to dismantling” [(16); see also (17–19)]. As Spicer writes (2019), “what was supposed to be an invitation to collective survival becomes yet another form of individualism” even though, for Lorde, “…self-care wasn't buying a candle, a new herbal tea, or any other form of consumerism. Self-care was both the sustenance that sustained her ability to enact change and was in itself a radical act” (20). Further, we must account for Lorde's examination of the “devastating effects of overextension”—pushing herself to her limits and past them in service of her work. Her caution points us not to her individual decisions on when and how to work, but to the broader social, cultural, political, and economic conditions in which she was located, which she was trying to transform, and which contributed to her deterioration (16).

## The normalization of overwork, sport coaching, and wellness privilege

Many sociologists have written on the normalization of overwork—the acceptance and glorification of excessive work hours and intense workloads as a norm [e.g., (21)]. Overwork is normalized in the name of capitalist economic growth and profit, and thrives against the backdrop of neoliberalism and meritocracy as notions of self-worth and good character are tethered to demonstrations of hard work, dedication, achievement, competition, unlimited availability, and constant connectivity (22).

Scholars exploring different facets of sport work, including sport coaching, have also examined the normalization of overwork (including, but not limited to, sport coaching as precarious labour) and its negative health consequences [e.g., see (23–26)]. Sport coaching, as a profession in Canada, is unregulated, requiring coaches (volunteer or otherwise) to navigate the vast and messy domain of work that often necessitates going above and beyond traditional coaching roles. Such work may include managing athletes/teams (as well as parent expectations), administrative tasks, supporting and mentoring coaching staff, fundraising, and, in some cases, teaching courses (24). Further, female coaches are also expected to engage in emotional labour practices, such as managing athletes' feelings and experiences as they navigate performance pressures (24). Juggling the multitude of tasks characteristic of sport coaching in the Canadian context is certainly considered overwork and overextension.

Coaches' expectations to care for athletes, etc. in the ways mentioned above illuminates the unregulated and precarious work of sport coaches in the Canadian context. And yet, even though care and caring has received considerable uptake in sport and sport coaching scholarship (27–31), a common thread that runs through the sport system as it relates to sport coaches is that they are not considered subjects in need of care themselves as much as they are providers of care to athletes to ensure athletes are unharmed, have positive sport experiences, and perform well (32).

This was particularly evident in the first author's doctoral study examining notions and experiences of care in a youth competitive sport setting (33). While a few parent participants in the study commented on the lack of compensation for coaches going “above and beyond” in their efforts to help improve their child's sport skills and performance, coaches themselves were not considered as needing care. Rather, study participants (including the coaches themselves) understood coaches as the ones responsible for caring for, and attending to, the needs of athletes (and, by extension, athletes' parents) without any concern for their own care. This study also examined organizational and policy documents from the Ontario Volleyball Association (OVA) where care was constructed primarily around the prevention of athlete harm with detailed descriptions of prohibited and outlawed behaviours for coaches to avoid (34). There were no documents or training resources that considered care as anything other than the absence of harm, and coach study participants routinely noted that they were on their own in figuring out how actualize care and caring relations in real life (35).

The issue of coaches' care is also absent from the Coaching Association of Canada's (CAC) National Coach Certification Program (NCCP). Of the 37 multi-sport modules offered through the NCCP (36), only one module, *Mental Health in Sport*, makes specific reference to coaches' well-being; the course objective reads: “to educate coaches about mental health to empower them to effectively play a role in supporting the well-being of the participants in their sport program, while also supporting their own mental health” (37). Two other modules, *Make Ethical Decisions* and *Managing Conflict*, allude to organizational care for coaches in their descriptions; however, athletes are still the primary focus with coaches' care being necessary only as a means to an end. For example, in the *Managing Conflict* module, “coaches will be able to listen and speak for themselves in conflict situations to maintain positive and healthy relationships with athletes, parents, guardians, officials, other coaches, and administrators” (38).

The Canadian sport system is dependent on individuals behaving in caring ways towards one another and on governing bodies creating and sustaining conditions conducive to caring relations (39, 40). The narrow understanding of who needs care and lack of effective organization around the care needs of all participants has not gone unnoticed by sport scholars, especially in the high-performance sport context (7). Coaches are expected to manage intense workplace stress, complex relationships with others, and performance pressures while operating effectively [e.g., (41)]—that is, while “charged with the care, development, and success of numerous athletes, support staff, and wider organizational personnel” (7). What has not yet coalesced is an outcry from the Canadian sport and sport coaching communities vocalizing that caring for coaches is caring for athletes.

Lorde invites readers to see self-care not as a personal coping strategy to maintain our regular routines, but as an act of resistance against structural inequities that are easily obscured as individuals' problems (42). By applying a political lens to care, she carves open a space for us to see that wellness is a resource that not all individuals enjoy equally [cf., (43)]. Wellness privilege refers to the advantages and benefits that certain individuals and groups experience in terms of their ability to access, and engage in, activities and practices that promote physical, mental, and emotional well-being (17). As Wakefield and Cole (44) note:

A political lens on care recognizes that some of us have the privilege of being cared for, while some of us primarily care for others. Gender, race, and class impacts all our experiences of care. The care we give or receive depends, in part, on our place in various hierarchies and the influence of dominant ideologies. Inequalities of power are built into organizational systems, processes, and expectations, which means that well-being privilege becomes invisible. (para. 10)

The ways in which care and caring are conceptualized and practiced in the existing sport system renders wellness privilege invisible, individualized, and depoliticized [cf., (45)]. This is profoundly problematic for sport coaches when held alongside the normalization of their overwork and the often (but not always) unspoken expectation that they give all of themselves for the betterment of their athletes. With the heightened focus on safe sport, the increased call on coaches to ensure that athletes are cared for is not being matched by increased attention and resources for the care and support of coaches (the carers) by sport organizations. We fear that efforts towards ensuring athletes are protected will be ineffective if care as a fundamental requirement for safe sport is not extended to encompass sport coaches too, as there is no shortage of narratives from coaches themselves where their vulnerability, their stress, their exhaustion, their need for care is hidden in efforts to be understood as effective and credible in their roles [see (41, 46, 47)].

## Where do we go from here?

It is not enough for us to encourage coaches to preserve themselves. Coaches' wellness is compartmentalized within the sport system as something separate from that of athletes’ wellness, as something that coaches need to attend to on their own time, with their own resources (financial or otherwise), and without disrupting the sport performance imperative (6). Sport and sport coaching leaders, including governing and educational bodies, play a significant role in mobilizing change as, “care happens more easily when it is wholeheartedly supported by leaders” and when, “our work cultures, relationships, and environments are aligned with…values…[of] reciprocity, mutuality and solidarity” (44). Better valuing coaches' work (including fair compensation and appropriate recognition across the sport system); refusing to reward overwork or overextension; recognizing that stress, anxiety, and burnout are not inevitable in sport coaching; providing meaningful care support to coaches without cost; doing justice to the concept of work-life balance for coaches—these are but a handful of measures that are available to sport leaders and organizations to cultivate self and collective care in sport and we call upon them to acknowledge and change the current systems to care for the carers.

Returning to Lorde's words, self-care is not self-indulgence but, instead, an act of self-preservation. If coaches are constantly working within a system that requires them to overwork and overextend themselves in the name of athlete wellness and performance, self-care would certainly be a political act—one that ought to provoke the inclusion of collective care into the policies and structures of sport organizations. We call upon the leaders of these organizations to make these changes not only to ensure safe sport practices, but for the betterment of sport for all who participate.
